# Cellular and Oxidative Mechanisms Associated with Interleukin-6 Signaling in the Vasculature

**DOI:** 10.3390/ijms18122563

**Published:** 2017-11-29

**Authors:** Sean P. Didion

**Affiliations:** Departments of Pharmacology and Neurology, The University of Mississippi Medical Center, Arthur C. Guyton Laboratory Research Building, Jackson, MS 39216, USA; didionlab@gmail.com; Tel.: +1-601-984-1710; Fax: +1-601-984-1637

**Keywords:** angiotensin II, endothelial nitric oxide synthase, inflammation, NADPH oxidase, superoxide, vascular biology

## Abstract

Reactive oxygen species, particularly superoxide, promote endothelial dysfunction and alterations in vascular structure. It is increasingly recognized that inflammatory cytokines, such as interleukin-6 (IL-6), contribute to endothelial dysfunction and vascular hypertrophy and fibrosis. IL-6 is increased in a number of cardiovascular diseases, including hypertension. IL-6 is also associated with a higher incidence of future cardiovascular events and all-cause mortality. Both immune and vascular cells produce IL-6 in response to a number of stimuli, such as angiotensin II. The vasculature is responsive to IL-6 produced from vascular and non-vascular sources via classical IL-6 signaling involving a membrane-bound IL-6 receptor (IL-6R) and membrane-bound gp130 via Jak/STAT as well as SHP2-dependent signaling pathways. IL-6 signaling is unique because it can also occur via a soluble IL-6 receptor (sIL-6R) which allows for IL-6 signaling in tissues that do not normally express IL-6R through a process referred to as IL-6 trans-signaling. IL-6 signaling mediates a vast array of effects in the vascular wall, including endothelial activation, vascular permeability, immune cell recruitment, endothelial dysfunction, as well as vascular hypertrophy and fibrosis. Many of the effects of IL-6 on vascular function and structure are representative of loss or reductions in nitric oxide (NO) bioavailability. IL-6 has direct effects on endothelial nitric oxide synthase activity and expression as well as increasing vascular superoxide, which rapidly inactivates NO thereby limiting NO bioavailability. The goal of this review is to highlight both the cellular and oxidative mechanisms associated with IL-6-signaling in the vascular wall in general, in hypertension, and in response to angiotensin II.

## 1. Linking Oxidative Stress with Hypertension and Vascular Dysfunction

It is generally well accepted that oxidative stress plays an important role in hypertension and related vascular sequelae, including endothelial dysfunction and vascular hypertrophy and fibrosis [1,2,3,4]. Oxidative stress is defined as an increase in reactive oxygen species, such as superoxide, due to increased production or reduced metabolism. Increases in vascular superoxide have several negative consequences on vascular biology, including reductions in nitric oxide (NO) bioavailability. NO, especially that derived from endothelial nitric oxide synthase (eNOS), normally exerts many protective effects on the vessel wall such as limiting endothelial activation, inflammation, vascular growth, and fibrosis [5,6,7,8,9]. Nitric oxide derived from the enzymatic activity of eNOS also plays an important role in endothelial responses in numerous blood vessels [10,11,12,13]. eNOS expression and activity is regulated at the transcriptional and translation level as well as by multiple post-translational mechanisms, including acetylation, phosphorylation, and sumolyation [14,15,16]. In addition, increases in vascular superoxide can greatly limit NO bioavailability thus negatively impacting endothelial function.

Some of the first evidence to implicate superoxide in the impairment of NO-mediated endothelial function came from studies in which exogenous superoxide dismutase, a scavenger of superoxide, was demonstrated to improve endothelial function in hypertensive blood vessels [17]. Infusion of exogenous SOD also was found to lower blood pressure in spontaneously hypertensive rats implicating a role for superoxide in hypertension [18,19]. Thus, pharmacological interventions aimed at reducing superoxide result in increased NO bioavailability, blood pressure lowering, and improvement of endothelial function.

Since these initial findings, a number of pharmacological and genetic approaches have provided additional evidence implicating an important role for oxidative stress, particularly NADPH oxidase-derived superoxide, in the development of hypertension and vascular dysfunction [20,21,22,23,24,25,26,27,28,29,30,31]. Increases in vascular superoxide may be an important link between activation of inflammatory molecules and pathways and endothelial dysfunction in cardiovascular disease. Emerging evidence suggests inflammatory cytokines, such as interleukin-6 (IL-6), affect expression and activity of both eNOS and NADPH oxidase thereby influencing NO and superoxide levels and contributing to oxidative stress [32,33,34,35,36,37]. The identification of vascular oxidases distinct from that expressed in neutrophils and macrophages was a major advance in the field of vascular biology [38,39]. A seminal discovery in this regard was the finding that angiotensin II stimulated NADH and NAPDH oxidase activity in cultured vascular smooth muscle [38]. Subsequent studies identified several isoforms of Nox, the main catalytic NADPH oxidase, and accessory proteins, such as p22phox, p47phox, p67phox and Rac1, which enhance oxidase activity and superoxide generation. There are several excellent reviews on the role of NADPH oxidase in the vasculature to which the author would refer the reader [40,41,42,43]. In addition to NADPH oxidase, other important enzymatic sources of superoxide in the vasculature include cyclooxygenase, cytochrome P450, uncoupled eNOS, and xanthine oxidase [44].

**Figure 1:**
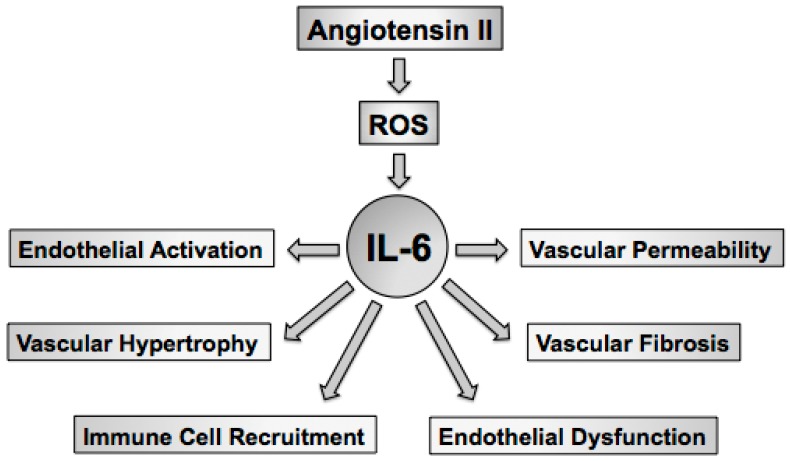
Vascular expression of IL-6, such as that produced by angiotensin II, is associated with alterations in vascular function and structure. Increases in reactive oxygen species, such as superoxide, serve to increase IL-6 expression in vascular cells. Increases in vascular or immune cell-derived IL-6 serve as a key mediator of several vascular phenotypes that are produced in response to angiotensin II. IL-6, interleukin-6; ROS, reactive oxygen species.

Hypertension or high blood pressure is the product of central, renal, and vascular mechanisms. Oxidative stress in the brain, kidney, and vasculature have been linked to hypertension as strategies aimed at lowering superoxide in all three organs has been shown to limit blood pressure and hypertension-related tissue injury [45,46,47,48,49,50,51]. The goal of this review is to highlight the effects of the proinflammatory cytokine IL-6 on oxidative and inflammatory mechanisms in general, in hypertension, and in response to angiotensin II [52,53,54,55,56,57,58,59,60,61]. As IL-6 has many important effects on blood vessels, including endothelial activation [62,63,64], immune cell recruitment [65,66,67,68,69,70], vascular permeability [71,72], vascular hypertrophy [73,74], and vascular fibrosis [75,76], and endothelial dysfunction [57,58] the discussion of this review will be limited to the effects of IL-6 on the vasculature (Figure 1).

## 2. Interleukin-6

IL-6 is a pleiotropic cytokine best recognized as a primary mediator of the acute phase response [77]. IL-6 also plays key roles in host defense, inflammation, cancer, as well as cellular growth and hypertrophy [78,79]. A number of stimuli, including angiotensin II as well as other inflammatory cytokines and growth factors, such as IL-1, TNFα and PDGF, are associated with increases in vascular cell-derived IL-6 [53,58,59,71,74,80,81,82,83,84,85,86,87,88,89,90,91,92,93,94,95,96,97,98,99,100,101,102,103,104,105,106,107,108,109,110,111,112,113,114,115] (Table 1). IL-6 levels are increased in cardiovascular disease, including atherosclerosis and hypertension, where IL-6 is thought to promote alterations in vascular function and structure [55,57,58]. For example, plasma IL-6 concentrations correlate with blood pressure, plasma angiotensin II levels, and vascular hypertrophy, suggesting an important role of IL-6 in the development and maintenance of hypertension, particularly that mediated by angiotensin II [58]. Thus, there is substantial data implicating IL-6 in the initiation as well as progression and maintenance of cardiovascular disease through reductions in NO, increases in vascular superoxide, and alterations in vascular function.

## 3. Interleukin-6 Signaling

**Figure 2:**
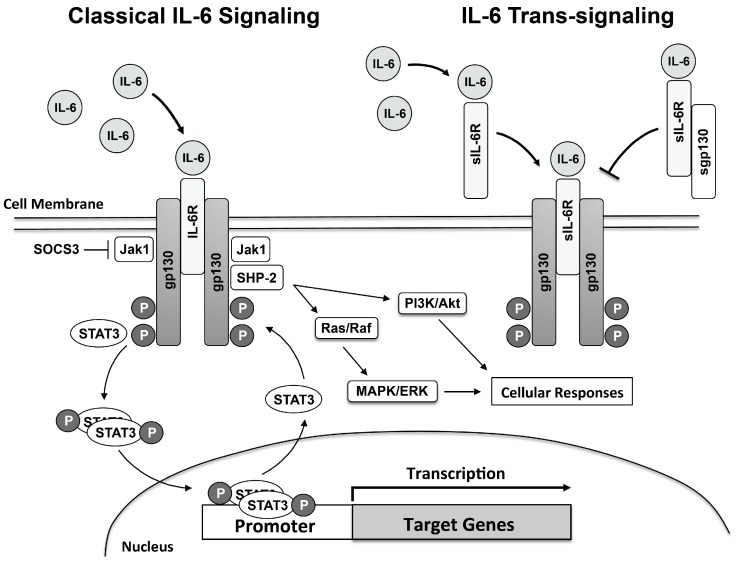
IL-6 signaling is a function of both classical IL-6 signaling and IL-6-trans-signaling. Classical IL-6 signaling involves IL-6 binding a membrane-bound IL-6 receptor (IL-6R), which facilitates dimerization of membrane-bound gp130. IL-6R lacks intrinsic signaling capability, however activated gp130 contains binding sites for recruitment and phosphorylation of signaling molecules such as Jak/STAT and SHP-2, which play important roles in transcription of IL-6 target genes, such as SOCS3. IL-6-trans-signaling occurs via IL-6 binding soluble IL-6 receptor (sIL-6R), which increases the half-life of IL-6 and serves as an important mechanism of IL-6 signaling in tissues that express gp130 but not IL-6R. Classical IL-6 signaling and IL-6-trans-signaling plays an important role in the diversity of IL-6 signaling. Expression of a soluble form of gp130 (sgp130) functions as an important endogenous inhibitor of IL-6 signaling by binding and preventing IL-6/sIL-6R complex binding and activation of membrane-bound gp130. Jak, Janus-associated Kinase; STAT, Signaling Transducer and Activator of Transcription; SOCS, Suppressor of Cytokine Signaling; PI3K, Phosphatidylinositol-4,5-bisphosphate 3-kinase; Akt, protein kinase B; ERK, Extracellular Signal-Regulated Kinase; MAPK, Mitogen-Activated Protein Kinase.

Although reviewed in greater depth in several excellent reviews on the subject, IL-6 signaling will be briefly discussed in terms of general mechanisms [117,118,119]. Classical IL-6 signaling involves IL-6 binding to its cognate membrane-bound IL-6 receptor (IL-6R) [78]. Expression of IL-6R is normally very low and expressed in a limited number of cell types, mainly immune cells and hepatocytes [118,119]. Although IL-6R has no intrinsic signaling capability interaction of the IL-6/IL-6R complex induces dimerization of the membrane-bound gp130 (the signaling molecule for IL-6R and related cytokine receptors) an important step in the transduction of the IL-6 signal [120,121,122,123]. Unlike IL-6R, gp130 is ubiquitously expressed and possesses intrinsic signaling capability. IL-6/IL-6R complex association and binding of gp130 initiates cellular signaling through several pathways including Janus-associated kinases (Jak; including Jak1, Jak2 and Tyk1), signal transducer and activator of transcription-1, -3, and -5 (STAT1, STAT3, and STAT5), SH2-domain containing phosphatase (SHP2), and extracellular signal related kinase 1/2, mitogen-activated protein kinase and phosphatidylinositol-3-kinase (PI3K/Akt) signaling pathways [78,119,124]. gp130-mediated activation of Jak family members and subsequent phosphorylation of specific tyrosine residues contained in the cytoplasmic portion of gp130, which serve as docking sites for recruitment of STAT molecules, results in phosphorylation and dimerization of phosphorylated STAT [78,124]. Phosphorylated STAT molecules migrate to the nucleus where they are involved in transcriptional activation of IL-6-dependent genes [124] (Figure 2).

In addition to classical IL-6R signaling, IL-6 signaling can also occur in non-IL-6R expressing cells via IL-6 trans-signaling involving a soluble IL-6R (sIL-6R) [124,125,126,127]. sIL-6R derived primarily from cleavage of membrane-bound IL-6R through ADAM17 and ADAM10 activation and to a lesser degree through alternate IL-6R splice variant [128,129]. ADAM17 and ADAM10 appear to promote release of sIL-6R in humans, whereas only ADAM10 has been found to be involved in production and release of sIL-6R in mice [129]. Binding of IL-6 to sIL-6R and subsequent binding to tissue bound gp130 IL-6/sIL-6R signaling transduction proceeds in a similar manner as classical IL-6R signaling (Figure 2). sIL-6R has the added advantage that it also serves to bind circulating IL-6 thereby greatly extending the half-life of IL-6 [130]. sIL-6R also regulates leukocyte interactions with endothelium, for example sIL-6R promotes endothelial production of monocyte chemoattractant protein-1 (MCP-1 aka CCL2), a key chemokine that regulates monocyte/macrophage infiltration to the vascular wall [64].

In order to limit the proinflammatory effects of IL-6, IL-6 signaling is regulated by a number of inhibitory molecules and processes. Inhibition of IL-6 signaling is important to limit inflammation and the untoward effects, such as tissue injury, associated with chronic IL-6 signaling. Therefore, IL-6 signaling is tightly regulated and highly specific and involves several different inhibitory mechanisms. For example, STAT3-dependent transcription is rapidly inactivated by suppressor of cytokine signaling 3 (SOCS3), which is itself an IL-6 target gene [131,132,133,134]. SOCS3 serves to functionally inhibit IL-6 signaling by binding to gp130 and Jak by targeting the complex for ubiquitination and degradation [132]. SOCS3 also interacts with SHP2 domain thereby reducing SHP2 signaling [132]. Thus, IL-6-induced SOCS3 expression serves as a major negative feedback loop for IL-6 signaling.

In addition to sIL-6R, a soluble form of gp130 (sgp130) has also been identified in plasma [135,136]. sgp130 serves as a functional antagonist of IL-6 trans-signaling by binding to the IL-6/sIL-6R complex thereby preventing the IL-6/IL-6R complex from binding with membrane-bound gp130 [137]. The antagonistic effect of sgp130 is greatly enhanced in the presence of sIL-6R and it has been postulated that IL-6R/sgp130 stoichiometry may be an important determinant of sIL-6R activity [138,139,140,141]. The potential therapeutic use of sgp130 has been exploited in the development of a recombinant form of sgp130, which contains an Fc moiety, sgp130-Fc [78,142].

A third mechanism of IL-6 signaling inhibition involves protein inhibitor of activated STAT-3 (PIAS3), which binds elements in IL-6 target genes thereby inhibiting phosphorylated STAT3/DNA interaction and gene transcription [142]. Whether PIAS3 is functionally important in the vasculature has yet to be determined. Nonetheless, exploitation of PIAS3 may represent a novel mechanism to limit IL-6 and IL-6-related injury in the vascular wall.

## 4. Vascular Sources of Interleukin-6

Several immune cell types, including macrophages, monocytes, B cells and T cells, produce IL-6 [125,143]. In addition, major components of the vascular wall, including endothelial cells, vascular smooth muscle cells, and fibroblasts are also important sources of IL-6 [92,93,94,95,96,97,98,144]. Thus, increased vascular expression of IL-6 most likely reflects that derived from both resident and infiltrating immune cells as well as vascular cells themselves. Increases in IL-6 originating within the vascular wall can act in either an autocrine or paracrine fashion that promote vascular dysfunction through increases in reactive oxygen species and reductions in NO which then serves to promote immune cell activation and vascular infiltration. Aging also appears to influence vascular IL-6 production as aged aortic vascular smooth cells exhibit higher basal secretion of IL-6 than young vascular smooth muscle cells [145]. Thus, vascular cells as well as resident and infiltrating immune cells have the capacity to produce IL-6 in response to a vast array of stimuli (Table 1). In addition, vascular cells are responsive to IL-6 via classical IL-6- as well as IL-6-trans-signaling, the latter of which appears to be the main mechanism of IL-6 signaling in vascular cells. Such a dual signaling pathway has been proposed to endow vascular and immune cells with specific cellular responses to various IL-6-producing stimuli [146]. The remaining discussion will be devoted to the effects of IL-6 on the three major vascular cell types followed by the effects of IL-6 on endothelial function and blood pressure.

## 5. Effect of Interleukin-6 on Endothelial Cells

Exposure of endothelial cells to hypoxia is associated with increases in oxidative stress and IL-6 as well as increases in endothelial cell permeability [59,71,72]. Anti-IL-6 neutralizing antibodies reduce both oxidative stress and endothelial permeability as measured by trans-endothelial electrical resistance, whereas addition of exogenous IL-6 has the opposite effect. Interestingly, antioxidants are very effective in limiting increases in reactive oxygen species, endothelial permeability, as well as IL-6 produced in response to hypoxia [59,71]. These results suggest that increases in reactive oxygen species, most likely functioning as signaling molecules, occurs upstream of both the increase in IL-6 and endothelial permeability produced by hypoxia.

Addition of IL-1 or IL-4 alone or in combination with INF-γ or TNF-α to cultured human aortic endothelial cells is associated with oxidative stress, as assessed using dihydroethidium staining, as well as marked increases in IL-6 expression [100]. Antioxidants or inhibitors of NADPH oxidase are very effective in limiting increases in IL-6 expression produced by IL-4 [100]. Consistent with these findings, IL-4 was unable to increase IL-6 levels in blood vessels from *Nox2*-deficient mice [100]. These findings provide pharmacological and genetic evidence linking NADPH oxidase-derived increases in oxidative stress and endothelial IL-6 production in response to inflammatory stimuli.

IL-6 can stimulate endothelial expression of adhesion molecules, including ICAM-1, VCAM-1 as well as E-selectin, thereby enhancing immune cell adherence and extravasation into the vascular wall [65,147,148]. For example, IL-6 has been shown to elevate ICAM-1 expression in endothelial cells via a STAT3-dependent mechanism involving both increases in oxidative stress and activation of Rac1 [148]. Reductions in NO bioavailability have been suggested to contribute in part to an increase in ICAM-1 expression as NO donors have been found to effectively suppress ICAM-1 expression and STAT3 phosphorylation in endothelial cells [147]. IL-6 mediated increases in adhesion molecule expression is thought to play an important role in the early phase of inflammation where lymphocytes such as neutrophils predominate, whereas in latter phases infiltration of monocytic cells, such as macrophages, predominate [67]. Indeed, IL-6 mediated reductions in NO bioavailability and increases in oxidative stress are associated with increased endothelial production of MCP-1 [149].

IL-6 is associated with dose-dependent reductions in eNOS expression in cultured endothelial cells [150,151]. The reduction in eNOS expression appears to occur independently of any effect on eNOS mRNA stability [150]. Instead, IL-6 appears to inhibit *eNOS* promoter activity via STAT3-mediated inhibition of SIE at amino acid residue-1024 [150]. In addition, IL-6 is also associated with reduced eNOS activity due in part to reductions in eNOS phosphorylation at Ser1177 (human eNOS), a phosphorylation site associated with enhanced eNOS activity [151]. The observed reduction in eNOS phosphorylation is reflective of reductions in upstream Akt activity, a key kinase involved with phosphorylation of eNOS at Ser1177. Thus, IL-6 appears to directly regulate eNOS expression and activity in cultured endothelial cells resulting in decreased NO bioavailability, which would be predicted to impact endothelial function in vivo [152].

IL-6 also inhibits eNOS activity through increases in expression of caveolin-1 [151]. Caveolin-1 binds eNOS through protein:protein interaction thereby effectively suppressing eNOS activity [151]. eNOS activity is suppressed by caveolin-1 as the eNOS:caveolin-1 complex is trafficked to caveolae rather than the cytosol where eNOS activity is most functional. The effect of IL-6 stimulated increases in caveolin-1 is not limited to functional suppression of basal eNOS activity but also includes suppression of agonist-, such as bradykinin-, induced increases in eNOS activity and NO levels [151].

Combined, it appears that IL-6 alone, or in combination with other inflammatory stimuli, can serve to promote and enhance not only endothelial permeability, but also recruitment and infiltration of the vascular wall by circulating immune cells, such as neutrophils, lymphocytes and macrophages, through upregulation of adhesion molecules and chemoattractants such as VCAM-1, ICAM-1 and MCP-1. Thus, endothelial recruitment of immune cells serve to enhance the level of inflammatory molecules. This is important as immune cells, such as neutrophils and macrophages, are also key sources of IL-6 and superoxide.

## 6. Effect of Interleukin-6 on Vascular Smooth Muscle

In addition to its many effects on endothelium, IL-6 has numerous effects on vascular smooth muscle, such as inducing proliferation, migration, and hypertrophy of vascular muscle [57,73,153]. A common mechanism linking IL-6 with vascular smooth muscle cell proliferation, migration, and hypertrophy is oxidative stress, specifically superoxide [56]. Angiotensin II is capable of producing increases in vascular superoxide in part through activation of the vascular NADPH oxidase [38,40,41]. Angiotensin II-induced increases in IL-6 can be inhibited by the addition of either Jak2, STAT1, or p47phox (an important component of vascular NADPH oxidase complex) antibodies [154]. Similarly, enhanced smooth muscle cell production of IL-6 in response to angiotensin II can be markedly reduced in the presence of DPI and AG490, pharmacologic inhibitors of flavin-containing enzymes such as NADPH oxidase and Jak2, respectively [154]. These findings are not limited to vascular smooth muscle as similar findings have been observed in renal mesangial cells, which share many properties and phenotypic markers as vascular smooth muscle [155,156,157]. Collectively, these studies suggest that increases in reactive oxygen species particularly increases in vascular superoxide occur upstream of angiotensin II-induced increases in IL-6 and involve NADPH oxidase-derived superoxide. Angiotensin II-induced increases in smooth muscle IL-6 levels were found to occur in an NFκB dependent manner [54,61]. Elevation of IL-6 produced by angiotensin II involve both activation and translocation of NFκB from the cytosol to the nucleus where it is involved in IL-6 gene transcription. These findings provide strong evidence linking angiotensin II signaling and NFκB pathways with increased IL-6 production in vascular smooth muscle.

Early studies found that IL-6 promotes both proliferation and migration of vascular smooth muscle cells [80,158,159]. In terms of proliferation, IL-6 is associated with increases in vascular smooth muscle cell number [98]. The increase in smooth muscle cell proliferation in response to IL-6 stimulation is associated with an increase in platelet-derived growth factor (PDGF, a potent inducer of cell growth) and incubation of vascular smooth muscle cells with anti-PDGF antibodies are very effective in limiting the increase in cell number produced by IL-6 [112,113]. In cell migration assays, IL-6 has been found to promote migration of vascular smooth muscle cells [159]. Inhibition of IL-6 signaling with IL-6 neutralizing antibodies is associated with reductions in cell migration. Interestingly, 12(S)HETE-induced migration can also be inhibited by neutralizing IL-6 antibodies, thereby implicating a role for IL-6 in 12(S)HETE-induced smooth muscle cell migration [80].

Consistent with the effects of angiotensin II on vascular smooth muscle in cell culture, angiotensin II is associated with vascular hypertrophy in vivo [57,65,73,160]. For example, angiotensin II infusion produces marked increases in vascular cross-sectional area, an index of hypertrophy whereas IL-6 deficiency limits the hypertrophic effect of angiotensin II [57]. Similarly, pharmacological inhibition of IL-6 signaling with small molecular inhibitors of STAT3 also result in marked attenuation of the hypertrophic response of the carotid artery to angiotensin II [160]. The reduction in hypertrophy, which occurs in response to IL-6 deficiency appears to occur independently of blood pressure, whereas the reduction in hypertrophy produced by STAT3 inhibitors may be blood pressure-dependent as STAT3 inhibition produces significant reductions in arterial pressure [160]. Taken together these findings suggest that angiotensin II-induced hypertrophy is dependent on IL-6 and downstream IL-6 signaling involving STAT3.

While such studies provide strong evidence linking angiotensin II and IL-6 with vascular hypertrophy, it was not known whether the effects of IL-6 were mediated by classical IL-6R signaling or those produced by IL-6-trans-signaling. An innovative study employing sgp130Fc (a soluble form of gp130 that binds IL-6/sIL-6R complexes and prevents them from signaling) inhibited the development of hypertension, but not vascular hypertrophy [73]. These findings indicate that vascular hypertrophy, but not hypertension, in response to angiotensin II occurs via classical IL-6 signaling. These findings are important in that they demonstrate that IL-6 can have differential effects depending on which IL-6R signaling pathway is activated.

## 7. Effect of Interleukin-6 on Fibroblasts

Increased expression of IL-6 has been implicated as a contributing factor in vascular fibrosis [75,76,144]. Fibrosis of blood vessels is associated with reductions in vascular compliance and is associated with arterial stiffness and hypertension [161,162,163]. Both cardiac and vascular fibroblasts stimulated with angiotensin II are associated with increases in oxidative stress and IL-6 production [61,164,165]. For example, when cardiac fibroblasts, which normally express little to no IL-6, are treated with angiotensin II they produce IL-6 in both a concentration- and time-dependent manner [165]. Interestingly, in studies of vascular wall explants, adventitia produces much more IL-6 than either endothelium or the medial wall, suggesting that adventitia is a prominent source of IL-6 in the vascular wall [166]. Moreover, when cardiac fibroblasts are co-cultured with macrophages, IL-6 production in response to angiotensin II treatment increases more than 10-fold compared to IL-6 levels when either cell type is cultured alone and then treated with angiotensin II [165]. These findings suggest that fibroblasts in the presence of macrophages, which is most likely indicative of the situation that occurs in vivo in response to angiotensin II or hypertension, synergize to enhance IL-6 expression. Incubation of fibroblasts from *IL-6*-deficient mice with angiotensin II is associated with negligible IL-6 production [153]. Treatment of fibroblasts with IL-6 results in increased αSMA, a marker of fibroblast differentiation, and collagen expression in wild-type, but not *IL-6*-deficient, fibroblasts [153]. Inhibition of Jak or ERK signaling is associated with reduced expression of αSMA in response to IL-6 in cardiac fibroblasts [164]. Incubation of fibroblasts with IL-6 has similar effects as angiotensin II, including increases in TGF-β signaling and type I collagen expression [75].

Taking the cultured fibroblast studies back to the whole animal, angiotensin II infusion in wild-type mice was found to produce marked increases in cardiac fibrosis, which was not present in angiotensin II-infused *IL-6*-deficient mice [164]. IL-6 is also associated with increased expression of TGF-β, a pro-fibrotic cytokine and a key mediator of fibrogenesis, as TGF-β drives collagen production and extracellular matrix remodeling [167]. Co-culturing fibroblasts and macrophages and treating them with angiotensin II is associated with increased αSMA and type I collagen expression as well as TGF-β and Smad3 phosphorylation (markers of fibroblast activation), effects which are absent in fibroblasts from *IL-6*-deficient mice [168]. Interestingly, *IL-6*-deficient mice are characterized by impaired wound healing, a process that involves fibroblasts, TGF-β signaling, and extracellular matrix remodeling [169].

Fibroblasts exposed to physiological concentrations of hydrogen peroxide, a reactive oxygen species formed from the metabolism of superoxide, display transient increases in intracellular calcium that precede increases in fibroblast-derived IL-6 [116]. Inhibition of intracellular calcium release with zestospongin, an antagonist of the inositol 1,4,5-triphosphate receptor and reticular calcium, results in lowering of peroxide-induced IL-6 expression, suggesting fibroblast IL-6 expression in response to peroxide is calcium-dependent [116]. In addition to peroxide, angiotensin II-induced superoxide formation is associated with cardiac fibrosis. Both candesartan, an AT1R antagonist and DPI an inhibitor of flavin-containing enzymes, including NADPH oxidase is associated with reduction in angiotensin II-induced fibrosis [61].

Evidence suggests a correlation between IL-6 levels and increases in MMP-2 and TIMP-1 and -2 expression [153]. *IL-6*-deficiency is associated with alterations in MMP-2, TIMP-1 and TIMP-2 [153]. TIMPs are endogenous inhibitors of MMPs. IL-6 treatment reduces TIMP-2 expression in cultured fibroblasts. Interestingly, IL-6-induced inhibition of TIMP-2 expression may serve to promote angiogenesis, an effect that is absent in *IL-6*-deficient mice [153].

Dermal fibroblasts produce very little IL-6 when cultured as monolayers, but when seeded onto a collagenous matrix IL-6 expression increases more than 10-fold [144]. In addition to collagen, dermal fibroblasts respond to inflammatory cytokines such as IL-1 with increased production of IL-6. Incubation of primary human aortic adventitial fibroblasts with angiotensin II is associated with increased IL-6 and MCP-1 expression and monocyte recruitment, which activates fibroblast proliferation and adventitial thickening thereby creating a fibroblast-monocyte amplification loop [66,71]. Infusion of angiotensin II is similarly associated with marked increases in aortic IL-6, vascular superoxide, MCP-1 expression, and vascular monocyte/macrophage accumulation and activation in wild-type, but not *IL-6*-deficient, mice [71]. Interestingly, chronic infusion of supraphysiological concentrations of angiotensin II is associated with the development of aortic aneurysms and dissection, in essence a hyper-amplification of the fibroblast-monocyte/macrophage loop [71].

## 8. Effects of Interleukin-6 on Endothelial Function and Oxidative Stress

Although IL-6 is a pro-inflammatory cytokine, IL-6 has been found to have little to no effect on endothelial function [57,170]. For example, acute incubation (<22 h) of blood vessels with IL-6 has no apparent effect on responses to either acetylcholine or nitroprusside in carotid artery from healthy wild-type mice [57]. The lack of effect of IL-6 on endothelial function is consistent with IL-6 infusion having no effect on arterial pressure as discussed in the following section. The fact that IL-6 does not affect endothelial responses is somewhat surprising, as IL-6 has been shown to produce dose-dependent reductions in eNOS expression in cultured endothelial cells [150,157]. The reduction in eNOS produced by IL-6 reflects a decrease in steady-state levels of both eNOS message and protein [150]. The effect of IL-6 on eNOS expression appears to be mediated by increased STAT3-mediated inhibition of the *eNOS* promoter [150]. While there is some evidence to suggest that human endothelial cells express IL-6R, it is not clear to what level IL-6R is expressed in vivo under basal conditions. Low or negligible IL-6R expression may explain, in part, why IL-6 infusion has been found to have little effect on vascular responses in vivo in the absence of disease. Future studies are required to delineate levels of endogenous IL-6R expression and IL-6R expression in response to IL-6 infusion.

Despite the effects of IL-6 on endothelial function and eNOS expression and activity in wild-type mice, IL-6 was observed to significantly impair endothelial responses in vessels with inherent reductions in *eNOS* expression [152]. For example, despite a 60% reduction in eNOS expression endothelial responses to acetylcholine are normal in *eNOS*^+/−^ mice. Incubation of vessels from *eNOS*^+/−^ with IL-6 however produces marked impairment of endothelial responses to acetylcholine [152]. IL-6-mediated impairment of endothelial function in *eNOS*^+/−^ mice is associated with increases in NADPH-stimulated superoxide levels, suggesting that IL-6-mediated increases in NADPH oxidase activity are greater in the absence of a single *eNOS* gene [152]. These data suggest that (1) despite marked reduction in eNOS expression, blood vessels such as carotid artery maintain their dependence on eNOS and NO in the absence a single *eNOS* gene, and (2) NADPH oxidase is an important source of vascular superoxide in response to IL-6 and that in the absence of a single *eNOS* gene the effect of IL-6 on NADPH oxidase activity is greatly enhanced. These findings may explain why IL-6 infusion is associated with increased arterial pressure and enhanced vascular contraction in pregnant but not non-pregnant rats, where eNOS expression may be altered in the former [171,172]. Taken together, such findings suggest that the effect of IL-6 on endothelial function only becomes apparent with marked reductions in eNOS expression (≥60%) such as that associated with heterozygous *eNOS* deficiency, *eNOS* polymorphisms, or cardiovascular risk factors associated with reduced eNOS expression.

Angiotensin II is elevated in several diseases, including atherosclerosis and hypertension [41,173,174]. Angiotensin II promotes increases in superoxide and a particularly important source of superoxide in response to angiotensin II involves NADPH oxidase [53,56,57]. NADPH oxidase expression increases in response to angiotensin II via an AT1 receptor dependent mechanism, as angiotensin II receptor blockers and AT1R deficiency are associated with reductions in angiotensin II-induced superoxide production [56]. IL-6 promotes increases in angiotensin II receptor expression further promoting increases in angiotensin II-induced superoxide. Angiotensin II stimulated increases in vascular superoxide is a key activator of NFκB-dependent IL-6 transcription. Similarly, IL-6-induced increases in vascular superoxide is dependent on Nox2 expression [53]. Thus, angiotensin II elevates superoxide levels, which leads to increases in IL-6 and IL-6-induced increases in Nox2-derived superoxide providing a vicious cycle promoting elevations in IL-6 and superoxide in the vascular wall. The increases in IL-6 and superoxide have been shown to impact both vascular function and vascular hypertrophy [53].

IL-6 has been shown to up-regulate AT1 receptor expression, leading to even greater levels of superoxide [56]. Angiotensin II impairs endothelial function via increases in vascular superoxide and reductions in nitric oxide bioavailability and deficiency of *IL-6* protects against angiotensin II-induced endothelial dysfunction and increases in vascular superoxide [57]. In reconstitution studies, incubation of vessels from *IL-6*-deficient mice with recombinant IL-6 recapitulates the impaired vascular phenotypes produced by angiotensin II, providing strong and convincing evidence that IL-6 is a critical mediator of angiotensin II-induced endothelial dysfunction [57]. Moreover, NADPH oxidase appears to be a major source of vascular superoxide and deficiency of *Nox2* (a major catalytic NADPH oxidase subunit) also protects against angiotensin II-induced impairment of endothelial function [57]. Incubation of vessels from *Nox2*-deficient mice with angiotensin II or recombinant IL-6 is not associated with endothelial dysfunction thereby strongly implicating Nox2-derived superoxide as an important vascular source of superoxide in response to angiotensin II and IL-6 [57]. Thus, angiotensin II is an important stimulus that increases vascular superoxide and vascular IL-6 expression, with increases in vascular superoxide being necessary for increased IL-6 expression presumably through activation of redox sensitive transcription factors such as NFκB [53].

## 9. Interleukin-6 and Blood Pressure

Early data from the literature suggested an association between blood pressure and circulating IL-6 levels in otherwise healthy men with IL-6 levels correlating in a linear fashion with every 10-mmHg increase in blood pressure [173]. Since this initial observation, several other studies provide additional support for increased IL-6 expression in hypertensive patients as well as several experimental models of hypertension [174,175,176]. Taken together, the clinical data links increased plasma IL-6 with disease progression. While such data provides evidence supporting an association between blood pressure and IL-6 it does not specifically demonstrate causality.

Several important questions regarding the role of IL-6 in blood pressure remain unanswered, such as what role does IL-6 levels play in maintenance of blood pressure in apparently healthy individuals. It is not clear whether the observed increase in IL-6 is due to increases in blood pressure or vice versa. Additionally, cardiovascular diseases associated with increases in IL-6, such as atherosclerosis and hypertension, are also associated with oxidative stress, particularly increases in vascular superoxide, suggesting a potential relationship between superoxide, IL-6 and arterial pressure.

One way to determine whether IL-6 affects blood pressure directly is to infuse IL-6. Interestingly, infusion of IL-6 at physiological concentrations associated with pathological conditions, but lower than that observed in sepsis, has little to no effect on arterial pressure when infused acutely or chronically [177,178,179]. (Table 2) The fact that IL-6 has little effect on blood pressure suggest IL-6 alone is not sufficient to affect blood pressure and that perhaps IL-6 requires activation of other inflammatory pathways in order to affect arterial pressure. Despite the lack of effect of IL-6 on arterial pressure, IL-6 is associated with a significant degree of cardiac fibrosis and hypertrophy, suggesting that IL-6 can produce cardiac alterations independent of changes in arterial pressure [179].

*IL-6*-transgenic mice are characterized by elevated plasma IL-6 levels as well as a number of outward phenotypes, including pulmonary hypertension [180,181]. Surprisingly, there have been no reported measurements of arterial pressure in *IL-6*-transgenic mice, perhaps due to the fact that transgenic expression of IL-6 is associated with enhanced mortality [180,182,183]. Generation of either vascular or immune cell specific *IL-6*-transgenic mice would be extremely helpful in determining the long term consequences, if any, associated with IL-6 overexpression on arterial pressure.

At the other end of the spectrum, *IL-6*-deficient mice have been used in a number of studies examining the effect of *IL-6*-deficiency on both baseline blood pressure and on the development of hypertension [57,73,81,163,184,185,186,187,188,189,190]. Baseline blood pressure in *IL-6*-deficient mice does not appear to be associated with alterations in arterial pressure, a consistent finding among several studies (Table 2). Such genetic findings provide strong evidence that IL-6 per se does not contribute to regulation of basal blood pressure. These findings are consistent with the concept that IL-6 expression would be negligible or minimal in the absence of disease.

While there is a clear consensus on the effect of IL-6 deficiency on baseline blood pressure, the effect of *IL-6*-deficiency on the development of hypertension is much more varied. For example, *IL-6*-deficiency has been associated with either no effect as well as partial inhibition of hypertension in various experimental models, such as that produced by angiotensin II-infusion alone or in combination with high-salt (Table 3). This is somewhat surprising as IL-6 levels are increased in response to angiotensin II infusion, a highly consistent finding among these same studies [186,188]. More recently, our laboratory found a direct correlation with plasma IL-6 levels and hypertension produced by angiotensin II infusion over a wide range of doses as well as with plasma angiotensin II levels [58].

Such observed differences in the effect of *IL-6*-deficiency on hypertension is most likely reflective of differences in the model of hypertension employed, such as angiotensin II-induced versus stress-induced hypertension, as well as differences in methods used to measure blood pressure (tail-cuff plethysmography versus radiotelemetry) [57,184,185,186,187,188,189,190]. Even when similar methods are used to produce hypertension and measure blood pressure, such as radiotelemetry, the gold standard for long-term and direct measurement of arterial pressure, major differences remain [184,186,190]. Thus, additional studies are needed to more clearly define the role of IL-6 in the development and maintenance of the hypertensive phenotype.

An important and most likely under reported event associated with angiotensin II infusion in C57Bl/6 mice, is the moderate incidence (≤30% incidence, depending on dose) of abdominal aortic aneurysm formation and rupture [191,192]. Some conflicting effects of IL-6 deficiency on blood pressure may be influenced by changes in aortic structure and death associated with aneurysm rupture such that a moderate degree of selection bias is introduced if the incidence of aneurysm is not considered or properly measured. Nonetheless, it is clear that IL-6 does not contribute to basal or resting blood pressure whereas IL-6 appears to contribute, in part, to the increase in blood pressure produced in experimental hypertension, including that produced by angiotensin II.

Examination of downstream mediators of IL-6 signaling, such as STAT3, has also revealed important insight into the role of IL-6 in experimental hypertension. For example, pharmacological inhibition of STAT3 with small-molecule inhibitors, such as S3I-201, which selectively inhibits STAT3 SH2-dependent complex formation, STAT3 dimerization and subsequent STAT3-dependent gene transcription, is associated with complete inhibition of angiotensin II-induced hypertension [160]. S31-201 also prevents angiotensin-II induced increases in vascular superoxide [160]. Interestingly, transgenic knockin mice expressing a functionally inactive STAT3 binding site on gp130, *S737A* mice, has been found to have no effect on baseline blood pressure and a normal pressor response to chronic angiotensin II infusion [193]. Whether endothelial responses and oxidative stress levels are altered in *S737A* mice, in general or in response to angiotensin II infusion, has not yet been determined. Taken together, these pharmacological and genetic data provide the first insights into downstream mechanisms of IL-6 signaling in angiotensin II-induced hypertension.

## 10. Summary

**Figure 3:**
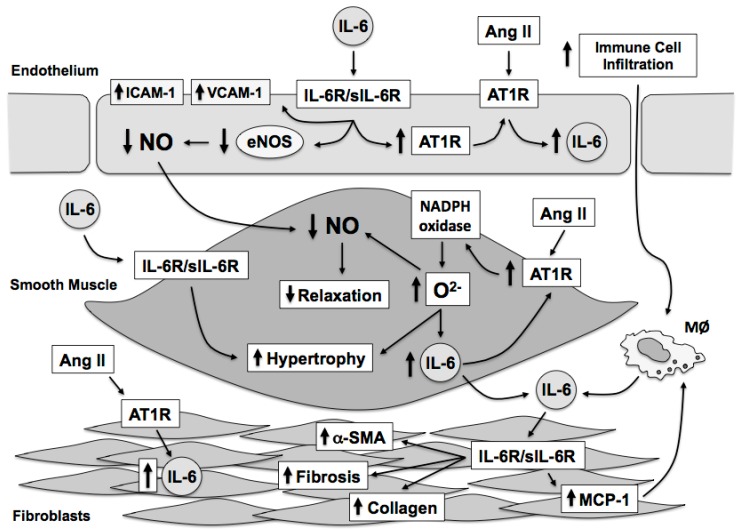
IL-6 signaling is associated with reductions in NO bioavailability, due, in large part, to reductions in eNOS expression and activity as well as increases in NADPH oxidase-derived superoxide. This combination promotes oxidative stress, endothelial dysfunction as well as vascular hypertrophy and fibrosis. Many of the effects of angiotensin II, such as increased vascular superoxide, are enhanced by IL-6 mediated increases in AT1 receptor expression. Similarly, angiotensin II and IL-6 synergize to promote increases in fibroblast SMA-α, collagen, and MCP-1 (CCL2) expression which promote matrix expansion that ultimately contributes to increased arterial compliance and vascular stiffness. IL-6 also promotes immune cell infiltration of the vascular wall through increased expression of adhesion molecules, such as ICAM-1 and VCAM-1, and expression of MCP-1. IL-6, interleukin-6; eNOS, endothelial nitric oxide synthase; AT1R, angiotensin type 1 receptor; ICAM, intracellular adhesion molecule; VCAM, vascular cell adhesion molecule; MØ, macrophage; SMA, smooth muscle actin; MCP-1, monocyte chemoattractant protein-1.

The relationship between angiotensin II, oxidative stress, and IL-6 is highly interdependent. Moreover, vascular cells can produce IL-6 in response to stimuli such as angiotensin II and IL-6 produced by vascular cells results in numerous and distinct effects on each vascular cell type (Figure 3). Angiotensin II increases IL-6 expression in the vascular wall through activation of the AT1 receptor and downstream activation of NADPH oxidase resulting in elevated oxidative stress, particularly superoxide that activates transcriptional mechanisms that directly promote IL-6 gene expression. Reciprocally, IL-6 promotes AT1 receptor expression further sensitizing the vascular wall to angiotensin II-dependent signaling mechanisms, which further promotes increases in oxidative stress and IL-6 expression. Vascular derived IL-6 plays an important role in the initiation of immune cell recruitment to the vascular wall. The increase in immune cells to the vascular wall serves to further promote endothelial dysfunction and recruitment of IL-6 and superoxide producing cells, such as neutrophils and macrophages. IL-6 is also associated with reductions in NO bioavailability through alterations in eNOS expression and activity. While IL-6 has many effects on vascular function and fibrosis, IL-6 does not appear to play a significant role in regulation of basal blood pressure. In contrast, IL-6 appears to contribute, in part, to the maintenance of the hypertensive phenotype as *IL-6*-deficiency is limits the rise in arterial pressure associated with some, but not all, forms of experimental hypertension. The role of IL-6 in hypertension development requires additional study in order define the specific contribution of IL-6 in the initiation and maintenance of the hypertensive phenotype. Nonetheless, IL-6 mediates many of the negative effects associated with hypertension and angiotensin II on vascular structure and function independent of blood pressure. While recent studies have begun to examine the role of downstream molecules, such as STAT3, in vascular function and oxidative stress, additional studies are required in order to provide a better understanding of the impact of IL-6 on both the cellular and oxidative mechanisms associated with IL-6 signaling in the vasculature.

## Figures and Tables

**Table 1:** Stimuli that increase IL-6 expression in vascular cells.

Stimulus	Reference
15(S)-HETE	[80]
Activated Protein C	[63]
Aldosterone	[81]
Angiotensin II	[52,53,58,74,83]
Endothelin	[84,85]
Fibrinogen	[86]
Histamine	[87,88]
Homocysteine	[89]
Hydrogen Peroxide	[61,116]
Hypoxia	[59,71,90]
Interleukin-1α/β	[94,95,96,97,98]
Interleukin-4	[99,100,101,102]
Interferon-γ	[98,103]
Lipopolysaccharide	[92,93,94,95,104,105,106]
Mechanical Stretch	[107,108,109,110]
Oxidized LDL	[111]
Platelet-derived Growth Factor	[112,113]
Serotonin	[114]
Thrombin	[86]
Tumor Necrosis Factor-α	[92,96,104,115]

**Table 2:** Effect of IL-6 infusion or *IL-6*-deficiency on Basal Blood Pressure.

Basal Blood Pressure		Reference
IL-6 Infusion	No effect	[177,178,179]
*IL-6*-Deficiency	No effect	[57,73,81,184,185,186,187,188,189]

**Table 3:** Effect of IL-6 Deficiency in Experimental Models of Hypertension.

Model	Hypertension Development	Reference
Acute Stress	Partial Inhibition	[185]
Ang II	No Effect	[57,185]
	Partial Inhibition	[73,187,188]
Ang II + High-salt	No Effect	[184,190]
	Partial Inhibition	[186]
Ang II + RKM	No Effect	[189]
DOCA-salt	No Effect	[81]
High-salt	No Effect	[184,186]

Ang II, angiotensin II; DOCA, deoxycorticosterone acetate-salt, RKM, reduced kidney mass.
